# Cone-beam computed tomography study of root and canal morphology of mandibular premolars in a western Chinese population

**DOI:** 10.1186/1471-2342-12-18

**Published:** 2012-07-20

**Authors:** Xuan Yu, Bin Guo, Ke-Zeng Li, Ru Zhang, Yuan-Yuan Tian, Hu Wang, Tao Hu DDS

**Affiliations:** 1 State Key Laboratory of Oral Diseases, Departments of Operative Dentistry and Endodontics, West China School of Stomatology, Sichuan University, Chengdu, P.R. China; 2 Institute of Stomatology of Chinese PLA General Hospital, Beijing, P.R. China; 3 Department of Radiology, West China School of Stomatology, Sichuan University, Chengdu, China; 4 Department of Stomatology, Yinzhou People's Hospital, Ningbo, Zhejiang, P.R.China

**Keywords:** Cone-beam computed tomography, Mandibular premolar, Morphology, Root canal configuration

## Abstract

**Background:**

Traditional radiography is limited in its ability to give reliable information on the number and morphology of root canals. The application of cone-beam computed tomography (CBCT) provides a non-invasive three-dimensional confirmatory diagnosis as a complement to conventional radiography. The aim of this study was to evaluate the root and canal morphology of mandibular premolars in a western Chinese population using CBCT scanning.

**Methods:**

The sample included 149 CBCT images comprising 178 mandibular first premolars and 178 second premolars. The tooth position, number of roots and canals, and canal configuration according to Vertucci’s classification were recorded.

**Results:**

The results showed that 98% of mandibular first premolars had one root and 2% had two roots; 87.1% had one canal, 11.2% had two canals and 0.6% had three canals. The prevalence of C-shaped canals was 1.1%. All mandibular second premolars had one root; 97.2% had one canal and 2.2% had two canals. The prevalence of C-shaped canals was 0.6%.

**Conclusions:**

The prevalence of multiple canals in mandibular first premolars was mainly of Type V, and mandibular second premolars had a low rate of canal variation in this western Chinese population. Root canal bifurcation occurred at the middle or apical third in most bicanal mandibular premolars. CBCT scanning can be used in the management of mandibular premolars with complex canal morphology.

## Background

A thorough knowledge of root canal morphology is essential for successful endodontic treatment. Neglecting to probe, prepare, and fill all of the canals can lead to failure of endodontic treatment [1]. As a group, the mandibular premolars are among the most difficult teeth to treat endodontically [2], because they have a high incidence of multiple roots or canals. A possible explanation for this difficulty may be the extreme variations in root canal morphology that occur in these teeth. Furthermore, the incidence, location, and morphology of root canal systems may vary in different ethnic or regional populations.

The dimensions of the mandibular premolar root canal system are wider buccolingually than mesiodistally. Two pulp horns are easily detected: a large, pointed buccal horn and a small, rounded lingual horn. At the cervix of the tooth, both the root and canal are oval; this shape tends to become flat or round where the canal approaches the middle of the root. If two canals exist, they are usually circular from the pulp cavity to their apical foramen. In another anatomic variation, a single, broad root canal may bifurcate into two separate root canals at the apex of the root [3]. Direct access to the buccal canal is usually possible, whereas the lingual canal is often very difficult to locate and tends to deviate from the main canal at a sharp angle. In addition, the lingual inclination of the crown tends to direct files buccally, making the location of a lingual canal orifice highly challenging [4]. A mandibular first premolar may sometimes have three roots and three canals [5-7] or one root and four canals [8]. One study reported a C-shaped canal anatomy in the mandibular first premolar [9].

Traditional radiography, hard tissue section, and root canal staining or micro-CT scanning *in vitro* are commonly used tools in identifying the configuration of canals. Conventional images compress three-dimensional (3D) anatomy into a two-dimensional image, resulting in some important features of the tooth and its surrounding tissues being visualized only in the mesio-distal plane. Thus, features presenting in the buccolingual dimension may not be fully appreciated. Cone-beam computed tomography (CBCT) scanning was introduced in the field of endodontics in 1990 [10]. This non-invasive, 3D imaging technique has many endodontic applications, including morphologic analysis [11]. Several studies of root canal morphology in permanent maxillary and mandibular first molars have been performed using CBCT, and the reports revealed that the application of CBCT is advantageous in identifying variations in canal configuration [12-15]. Compared with the helical CT scanner, its major advantages are a substantial reduction in radiation exposure [16] and higher-quality image rendering for assessment of dental hard tissues [17]. Many studies of root and canal morphology in mandibular premolars have been conducted because these teeth present complex morphology that often complicates treatment [18,19]. However, most of these studies have been performed *ex vivo*[20,21] and involved complete destruction of the tooth during examination (hard tissue sections) or have acquired only two-dimensional anatomic information (traditional radiography). Thus, the current study was designed to test 3D CBCT imaging as a means of determining root and canal morphology in mandibular first and second premolars as an adjunct to clinical diagnosis and treatment planning.

## Methods

All experimental procedures in this study were approved by the West China Stomatology School ethics committee. The West China Hospital of Stomatology, located in Chengdu, functions as the clinical treatment center for oral diseases and maxillofacial surgery in the Western-China area This area include Sichuan, Yunnan, Guizhou, Tibet etc. provinces, and is the most concentrated area of ethnic minorities in china, there are 44 ethnic minority groups except the Han ethnic, which is the main group. All of the Western population belong to the Asian.We selected 149 CBCT images from the medical imaging center at the West China Hospital of Stomatology, between July 2009 and December 2010. All images were taken using a 3D Accuitomo CBCT machine (MCT-1[EX-2 F], Morita Manufacturing Corp, Kyoto, Japan) with image capture parameters set at 80 kV and 5.0 mA, and an exposure time of 17 s. The voxel size was 0.125 mm and the slice thickness was 1.0 mm. Samples of fully erupted permanent mandibular first and second premolars were included. Qualifying mandibular premolars each demonstrated fully developed apices and lacked root canal fillings, posts and crown restorations. The CBCT images of 356 mandibular premolars from 149 patients of Chinese descent were analyzed with inbuilt software (i-Dixel one volume viewer 1.5.0) using a Dell Precision T5400 workstation (Dell, Round Rock, TX, USA). Axial, coronal, and sagittal two-dimensional sectional images were displayed on a 32-inch Dell LCD screen with a resolution of 1280 × 1024 pixels in a dark room. Two independent endodontists assessed the number of roots and canals, the position where canal bifurcation occurred and the canal configuration using One Data Viewer software (Morita Manufacturing Corp) to reach consensus in the interpretation of radiographic findings. In cases where consensus was not reached, a third professional oral radiologist was asked to perform a decisive evaluation.

## Results

### Number of roots and canals

Of 178 mandibular first premolars, 174 (98%) had one root and four (2%) had two roots; 87.1% had one canal, 11.2% had two canals, 0.6% had three canals and the prevalence of C-shaped canals was 1.1%. All mandibular second premolars had one root. Of these, 97.2% had one canal and 2.2% had two canals. The prevalence of C-shaped canals was 0.6% (Table 1).

**Table 1:** Number and percentage of roots and canals in 356 mandibular premolars according to location

	**No. of roots**				**No. of canals**							
	**One-rooted**		**Two-rooted**		**1**		**2**		**3**		**c-shaped**	
	**left**	**right**	**left**	**right**	**left**	**right**	**left**	**right**	**left**	**right**	**left**	**right**
First premolar	78	96	1	3	68	87	9	11	0	1	1	1
Total	174 (98%)		4 (2%)		155 (87.1%)		20 (11.2%)		1 (0.6%)		2 (1.1%)	
Second premolar	79	99	0	0	76	94	3	4	0	0	0	1
Total	178 (100%)		0 (0%)		173 (97.2%)		4 (2.2%)		0 (0%)		1 (0.6%)	

### Variations in root canal system morphology

**Figure 1:**
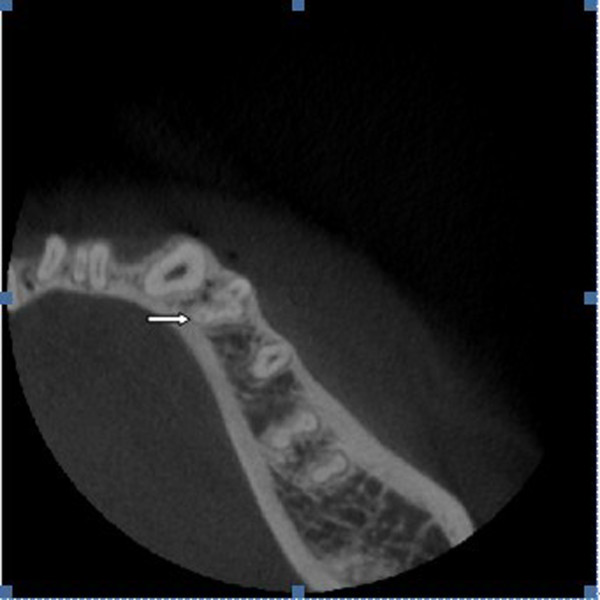
**Type VIII canal configuration in mandibular first premolar with three canals.** White arrow illustrates three independent canals in the cross-sectional image.

**Figure 2:**
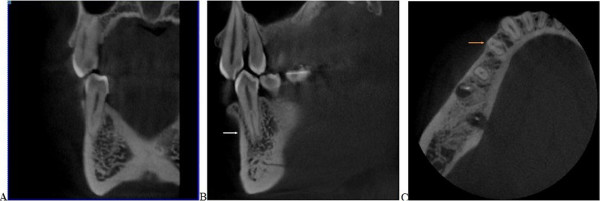
**Cross-sectional CBCT image of mandibular first premolar with a clearly distinguished C-shaped configuration**. Image **A** illustrates canal shape from the coronal aspect. White arrow denotes root canal in the sagittal plane (**B**), and the orange arrow denotes the cross-sectional image (**C**).

**Figure 3:**
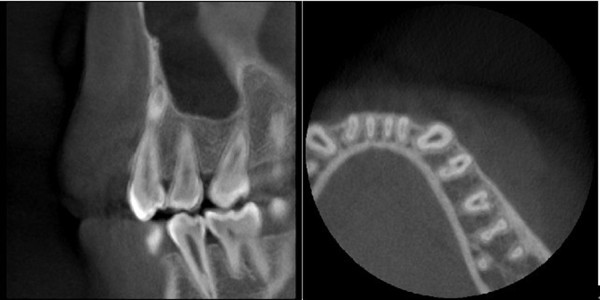
C-shaped canal configuration in a mandibular second premolar at the middle–apical part of the canal in cross-sectional view.

The canal morphology of mandibular first premolars according to Vertucci’s classification [22] was as follows: Type I = 151 (86.8%), Type III = 3 (1.7%), Type V = 17 (9.8%), Type VIII = 1 (0.6%) (Figure 1), while two teeth had a C-shaped configuration (1.1%) (Figure 2). Four of the mandibular first premolars had two roots and two canals (one canal in each root) (Table 2). All 178 mandibular second premolars were single-rooted and the canal configurations of these teeth according to Vertucci’s classification were Type I (173 teeth, 97.2%), Type II (one tooth, 0.55%) and Type V (3 teeth, 1.7%); one tooth had a C-shaped configuration (0.55%) (Figure 3, Table 3). In both first and second mandibular premolar teeth exhibiting Type V or Type VIII morphology, the canal bifurcation occurred at the middle-apical part of the root, which is where 87% and 75% of the canal system variations occur in mandibular first and second premolars, respectively.

**Table 2:** Number and percentage of canal system types in 174 single-rooted mandibular first premolars

**Root canal configurations**									
	**Type I 1**	**Type II 2-1**	**Type III 1-2-1**;	**Type IV 2**	**Type V 1-2**	**Type VI 2-1-2**	**Type VII 1-2-1-2**	**Type VIII 1-3**	**C-shaped**
Number	151	0	3	0	17	0	0	1	2
Percentage	86.8	0	1.7	0	9.8	0	0	0.6	1.1

**Table 3:** Number and percentage of canal system types in 178 mandibular second premolars

**Root canal configurations**									
	**Type I 1**	**Type II 2-1**	**Type III 1-2-1**	**Type IV 2**	**Type V 1-2**	**Type VI 2-1-2**	**Type VII 1-2-1-2**	**Type VIII 1-3**	**C-shaped**
Number	173	1	0	0	3	0	0	0	1
Percentage	97.2	0.55	0	0	1.7	0	0	0	0.55

## Discussion

Our findings on the number of roots and canals in mandibular first premolars, showing that most had only one root and one canal, are in keeping with the results of previous studies [18,22-25]. In mandibular second premolars, we also found that all had one root and most had only one canal, in agreement with the findings of Miyoshi et al. [26]. However, these results are somewhat different to those reported in other studies. One study of a Chinese population in the Taiwan area found that only 54% of mandibular first premolars exhibited a single canal, whereas 22% contained two canals (*vs.* 11% in this study) and 18% percent had a C-shaped configuration (*vs.* 1.1% in this study) [20]. Furthermore, a literature review showed that, on average, 91.0% of mandibular second premolars have a single canal and 9.0% have two or more canals (*vs.* 97.2% and 2.2%, respectively, in this study) [19]. These divergent results may be explained by methodological differences or by variations in sample size, ethnic and/or regional background of the samples used [9,18,20,27,28].

The cross-sectional morphology of the majority of canals in this study was oval in the coronal third, circular or oval in the middle third and circular in the apical third. Interestingly, the root canal bifurcation tended to occur in the middle or apical third in the vast majority of bicanal mandibular premolars (87% and 75% in first and second premolars, respectively), consistent with previous investigations [3,20,29]. This indicates a high probability of variation in the root canal when the clinician detects a change of shape or direction in the middle-apical sections of the canal.

The complex root canal anatomy of mandibular premolars may be disguised in routine straight-on or even oblique radiography in clinical situations. A previous study [21] has demonstrated the low sensitivity of mesiodistal or buccolingual angulated radiographs in detection of root canal morphology. Conventional intraoral periapical radiographs are an important clinical diagnostic tool for assessing canal morphology, but these radiographs are not completely reliable because of inherent limitations such as distortion and superimposition of dental structures [8]. The application of CBCT has been suggested in these cases to provide a 3D confirmatory diagnosis without causing any tooth damage. It offers high resolution and is well suited for endodontic applications as a complement to conventional radiography [14]. When uncertainty exists in the diagnosis of canal variations, or a change of shape/direction in the middle-apical third of the canal is detected, periapical radiography associated with CBCT can be used to determine or confirm the presence and location of canal bifurcation.

## Conclusion

Mandibular first premolars in a western Chinese population exhibited high variability and complexity in their canal systems. The root and canal configuration of mandibular second premolars was less variable than that of mandibular first premolars. Significantly, a CBCT scanner was able to detect these complex variations. This suggests that CBCT has potential as an auxiliary tool in the evaluation of mandibular premolars with complex canal morphology to improve the quality of root canal therapy. The importance of accurately determining the existence of complex canal systems is reflected in the elevated failure rate that occurs when additional canals are missed during root canal therapy. CBCT scanning is of great value in detecting anomalous canal morphology when diagnosis by conventional radiography is inconclusive.

## Competing interests

All authors declare that they have no competing interests.

## Authors’ contributions

XY, BG, RZ, KZL, YYT, TH and HW participated in the design of the experiment and wrote the manuscript. XY and BG participated in the acquisition, analysis and interpretation of data. All authors read and approved the final manuscript.

## Pre-publication history

The pre-publication history for this paper can be accessed here:

http://www.biomedcentral.com/1471-2342/12/18/prepub
